# Biological Sensors for Solar Ultraviolet Radiation

**DOI:** 10.3390/s110404277

**Published:** 2011-04-12

**Authors:** Teiti Yagura, Kazuo Makita, Hiromasa Yamamoto, Carlos F.M. Menck, André P. Schuch

**Affiliations:** 1 Department of Microbiology, Institute of Biomedical Sciences, University of São Paulo, São Paulo 05508-000, Brazil; E-Mails: teiti.yagura@usp.br (T.Y.); aschuch@usp.br (A.P.S.); 2 Faculty of Engineering, Takushoku University, Tokyo 193-0985, Japan; E-Mail: kmakita@la.takushoku-u.ac.jp; 3 Department of Physics, Rikkyo University, Tokyo 171-8501, Japan; E-Mail: yamamoto@rikkyo.ac.jp

**Keywords:** sunlight, UV radiation, biosensors, biological dosimetry, DNA damage

## Abstract

Solar ultraviolet (UV) radiation is widely known as a genotoxic environmental agent that affects Earth ecosystems and the human population. As a primary consequence of the stratospheric ozone layer depletion observed over the last decades, the increasing UV incidence levels have heightened the concern regarding deleterious consequences affecting both the biosphere and humans, thereby leading to an increase in scientific efforts to understand the role of sunlight in the induction of DNA damage, mutagenesis, and cell death. In fact, the various UV-wavelengths evoke characteristic biological impacts that greatly depend on light absorption of biomolecules, especially DNA, in living organisms, thereby justifying the increasing importance of developing biological sensors for monitoring the harmful impact of solar UV radiation under various environmental conditions. In this review, several types of biosensors proposed for laboratory and field application, that measure the biological effects of the UV component of sunlight, are described. Basically, the applicability of sensors based on DNA, bacteria or even mammalian cells are presented and compared. Data are also presented showing that on using DNA-based sensors, the various types of damage produced differ when this molecule is exposed in either an aqueous buffer or a dry solution. Apart from the data thus generated, the development of novel biosensors could help in evaluating the biological effects of sunlight on the environment. They also emerge as alternative tools for using live animals in the search for protective sunscreen products.

## 1. The UV Component of Sunlight

Ultraviolet (UV) radiation is part of the solar electromagnetic spectrum, with wavelengths shorter than those of visible light, but longer than X-rays. It is an essential factor for many global biological and environmental phenomena. There are three major subtypes of UV rays, namely, UVA (315–400 nm), UVB (280–315 nm) and UVC (100–280 nm).

UVA accounts for about 95% of the total UV energy that reaches the Earth’s surface, the remaining 5% being UVB. Seeing that the shorter the wavelength, the greater the absorption by the atmosphere, UVC, being totally absorbed by stratospheric gases, mainly oxygen and ozone, fails to reach the troposphere. Furthermore, since UVB is very effectively screened out by ozone molecules, only a small fraction actually reaches the surface, contrary to most of UVA. In the face of global efforts to diminish ozone-depleting substances, it can be said that, given the recent measures of increasing ozone levels worldwide [1], the Montreal Protocol on Substances That Deplete the Ozone Layer is really working.

**Figure 1.**
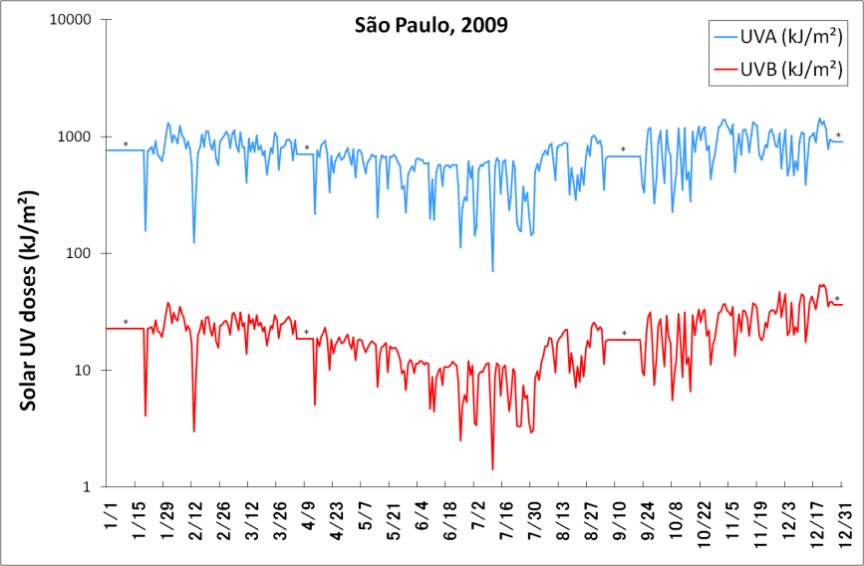
Year-round (2009) solar UVA (blue) and UVB (red) doses measured in São Paulo—SP (23°32′S, 46°38′W), Brazil. * Periods in which the measurements were not performed, due to technical reasons.

Furthermore, apart from the ozone-depleting gases policy, continuous efforts are under way to monitor the yearly incidence of surface UV radiation. Our research group has been dedicating special attention to the measurement of solar-UV rays in the city of São Paulo (23°32′S; 46°38′W), the largest in Brazil, and one of the most populous in the World. The incidence of solar UVB and UVA radiation has been measured throughout the day, over the last two years. In the year-round graph presented in Figure 1, the winter (June to August) reduction in UV levels (although lower than in higher latitudes) is more pronounced in UVB daily doses, mainly due to the solar-angle effect at this latitude, as UVB is more absorbed by the atmospheric air mass, whereas UVA practically freely passes through.

**Figure 2.**
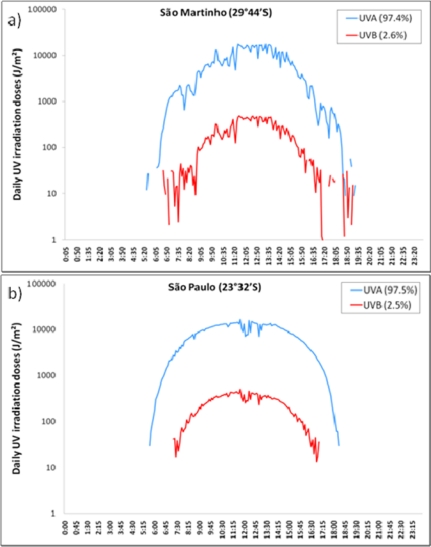

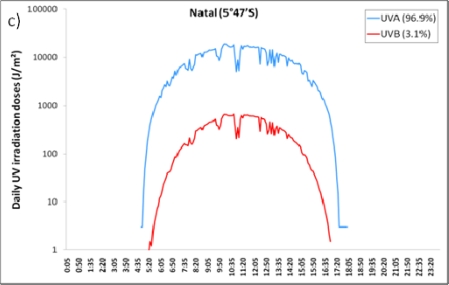
Solar UVA (blue) and UVB (red) irradiation profiles at **(a)** São Martinho da Serra—RS (29°44′S, 53°82′W), **(b)** São Paulo—SP (23°32′S, 46°38′W), and **(c)** Natal—RN (5°47′S, 35°12′W), Brazil.

Data of UVA and UVB doses for an entire day, at different latitudes in Brazil are presented in Figure 2 for comparison. The results show that the daily flow of UVA, besides being remarkably greater than UVB, is comparably more constant and detectable earlier in the day. Nevertheless, and as expected, at a lower latitude (Natal) UVB incidence is higher and can be detected earlier in the morning (around 6:00 a.m.), when compared to the other mid-latitudes (around 7:00 a.m.).

Ozone concentration, although important, is not the only factor exerting an influence on the incidence of UV radiation. The solar zenith angle, which varies according to the time of day, day of the year and latitude, also contributes enormously.

A further factor meriting consideration is the Earth’s elliptical orbit. As the Sun is on one of the foci of this ellipsis, this causes the Northern Hemisphere to be farther away from the sun in the summer in comparison to the Southern Hemisphere in the same season. Furthermore, other factors, some associated with anthropogenic activity, are capable of influencing UV incidence, *viz.*, air pollution/particulate matter emission, clouds (which can either diminish or increase UV irradiance), climate effects, albedo (the fraction of solar energy reflected from the Earth) and altitude [1,2].

Even though atmospheric ozone levels are recuperating, it remains uncertain whether climate change will delay or accelerate ozone recovery. As surface UV radiation levels continue on the rise, the consequential increase in risks involving both ecosystems and human health requires redoubled attention.

## 2. UV Effects on the Biosphere and Human Health

The biological consequences arising from increased UV irradiance are numerous. In terrestrial ecosystems, these affect plants, pathogens, herbivores, soil microbes and other basic processes. As each type of organism reacts to induced UV damage in a different manner, the eventual changes in balance can possibly lead to significant alterations in carbon and nitrogen cycling. Furthermore, apart from ozone concentration dependence, UV irradiance is also affected by climate change factors, thus complex interactions are expected to occur, thereby diversely affecting terrestrial ecosystems [3].

The effects of UV radiation on human health are better defined. Besides producing vitamin D, UVB radiation itself is correlated with skin cancer, photoaging, immunosupression and cataracts, to mention just a few of the harmful effects. It is widely known that in humans the most important benefit derives from the production of vitamin D. Nevertheless, there is a limit in this production, which, when passed, leads to the degradation of already formed vitamins, thereby attaining toxic levels, whereby the efforts concentrated on determining the “optimal level” of production. It has been shown that casual, and little daily UV doses are sufficient to prevent the lack of vitamin D [4]. However, there is evidence that modern lifestyles can be held responsible for the increasing levels of melanoma among indoor-workers. It is speculated that windows and sunscreens, which block mainly UVB and facilitate UVA penetration, give rise to a reduction in cutaneous vitamin D levels, possibly inversely correlated to the increase in the incidence of melanoma [5].

Mechanistically, UV irradiance is the cause of many deleterious effects, such as the induction of DNA damage, inseparable from those beneficial [6]. Furthermore, various UV wavelengths exhibit different skin-penetration capabilities, with diversification in carcinogenesis as the outcome [7].

Obviously, both ecosystems and the human population are always much more exposed to UVA than UVB irradiance, in absolute flow terms. Nevertheless, these values require weighting, using action spectra involving the relative biological effectiveness for various endpoints. With this in mind, knowledge on the UV pattern at different sites is of vital interest for determining the potential risks arising from local UV radiation worldwide. Thus, the development of appropriate biological sensors assumes an important role in a scenario of increasing UV incidence.

## 3. The DNA Molecule as the Main Target of UV Light in the Cells

**Figure 3.**
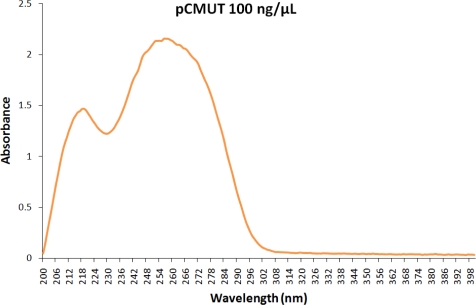
The absorption spectrum for the DNA molecule. A sample of purified plasmid DNA (pCMUT vector), diluted in a TE buffer (10 mM Tris-HCl [pH 8.0], 1 mM EDTA [pH 8.0]) at the indicated concentration, was used to obtain this spectrum, with an Evolution 300 UV-Vis Spectrophotometer (ThermoFisher Scientific, USA).

The most important cellular effects induced by UV radiation (cell-death and mutagenesis) are directly related to a chain of events that primarily involve the induction of DNA lesions. Notwithstanding, the chemical nature and efficiency in the formation of DNA lesions greatly depend on the wavelength of incident UV photons [6,8] as well as on the base composition of the DNA molecule, as previously demonstrated. In fact, the absorption spectra of DNA from various species for wavelengths greater than 300 nm clearly indicated that its relative absorption increases as a function of guanine-cytosine content [9]. Therefore, as the maximum of light absorption by DNA molecules is 260 nm, UVC is revealed as being the most effective wavelength for the induction of DNA photoproducts. The absorption spectrum of a purified plasmid DNA sample is presented in Figure 3, as a demonstrative example.

The different wavelengths of UV light induce different types of DNA damage [10]. The direct excitation of the DNA molecule by UV sunlight (mainly by UVB wavelengths) results in well-known modifications that trigger off dimerization reactions between adjacent pyrimidines. The main products resulting from these photochemical reactions are cyclobutane pyrimidine dimers (CPDs) and pyrimidine (6-4) pyrimidone photoproducts (6-4PPs) [6]. In addition, upon further irradiation with UVA wavelengths (around 320 nm), the normal isomers of 6-4 PPs can be converted to their Dewar valence isomers [11,12]. However, in certain dormant life-forms produced by bacteria, such as *Bacillus subtilis*, the only DNA photoproduct produced upon exposure to UV light corresponds to two thymines linked by the methyl group of one of the bases. The formation of this specific lesion, *viz.*, 5-thyminyl-5,6-dihydrothymine (spore photoproduct, SP), is possibly due to specific features of the spores, these including DNA conformation (A form), dehydration, the presence of dipicolinic acid in the core, and the binding of small acid-soluble proteins to DNA [13].

**Figure 4.**
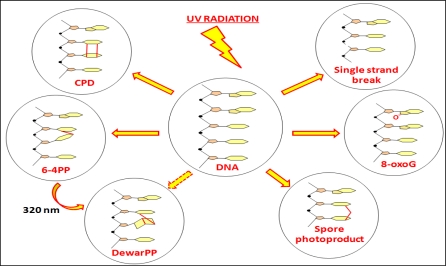
The main DNA lesions induced by UV light: CPD-cyclobutane pyrimidine dimer; 6-4PP-pyrimidine (6-4) pyrimidone photoproduct; DewarPP-Dewar valence isomer; Single strand breaks; 8-oxoG-7, 8-dihydro-8-oxoguanine; Spore photoproduct.

Apart from direct induction of DNA lesions, UV radiation can also cause DNA damage indirectly, following photon absorption by chromophores other than DNA itself, thereby generating reactive oxygen species [14]. Oxidatively generated DNA damage, mostly in the form of 7, 8-dihydro-8-oxoguanine (considered a marker for this type of damage), and which occurs more effectively with UVA than UVB, has often been proposed as a pre-mutagenic lesion in UVA mutagenesis [7,15–18]. Another type of UV-induced DNA lesion, although rather inefficiently so, is the single-strand break. It has also been suggested that this is probably an innocuous lesion with little involvement in the formation of mutations [6,19]. The main types of UV-induced DNA lesions are illustrated in Figure 4.

It is well-known that solar UV radiation can generate chemical modifications in the DNA structure, leading to several biological consequences. Thus, in the evolution of life on Earth, cells have developed specific DNA repair mechanisms capable of dealing with different types of lesions. In both prokaryotes and eukaryotes, these biochemical pathways are indispensable for maintaining genomic integrity by removing damaged DNA bases or short fragments of nucleotides containing UV photoproducts. However, through inadequate repair, unremoved UV-induced DNA damage possibly interferes with basic cellular processes, such as transcription and DNA replication, thereby leading to mutations and/or cell-death [20,21].

## 4. Biosensors for UV Light

In the 1980s, the discovery of a progressive decline in the stratospheric ozone layer and the consequential increase in UVB levels, aroused the interest of numerous research groups worldwide. There was a generalized attempt to evaluate the biological effects of solar UV radiation, through the development of dosimetric systems employing biological material [22,23]. In general, a biosensor integrates incident UV wavelengths of sunlight, thereby weighting them according to their respective biological effectiveness [24]. Hence, its spectral response is the related photobiological effect [25].

Over the latter decades, various simple test systems, such as provitamin D3 [26], uracil thin layers [24,27], DNA [6,28–30] or different bacteriophages [31,32], spores from *Bacillus subtilis* [23,25,33,34], and eukaryotic cells in culture [35], have been developed for use as biological UV dosimeters. Most of these tests reflect UV sensitivity of the main target of radiation in living organisms, by the direct or indirect measurement of DNA damaging capacity of solar UV radiation, as well as the initiating event in a variety of harmful effects to human health and life in general.

Considering that one of the most important criteria for the validity of a biosensor is the relevance of the respective photobiological/photochemical process, DNA-based biological dosimeters have a genuine biological appeal [25]. However, each type of biological material intended for use as a biological UV dosimeter needs to comply with several criteria, namely: (i) it should be clearly indicative of a certain biological effect induced by UV light that represents a possible risk or benefit to human health or ecosystems; (ii) the spectral response (UVB/UVA) should be in agreement with a specific photobiological process; (iii) quantification of the biological effects of UV light should be undertaken in measurable units; (iv) data should be reproducible; (v) the general requirements for radiometers (absolute response, linearity of response, angular response, and intercalibration with other biologically weighted spectroradiometers) should be complied with; (vi) the chosen biological system should be robust, with high resistance against changing environmental parameters, as temperature; (vii) suitability for routine measurement [22]. Below, features of the main biological models that have been developed for use as biodosimeters in the measurement of biological effectiveness of environmental UV radiation, will be described.

### 4.1. DNA Dosimetry

DNA, the genetic material of cells, is the main target molecule of UV radiation. As shown in Figure 3, this molecule possesses high sensitivity to short-wavelengths in the UV light spectrum (UVC > UVB > UVA), a feature that confers reasonable applicability for measuring the increasing incidence of solar UVB radiation, whence the various types of biological systems using DNA for evaluating the impact of UV light on the environment.

A UVB DNA-dosimeter was developed based on minidots of purified and dried (12–16 h at 40 °C) bacteriophage λ DNA placed on a UV transparent biofilm. In this system, photo-induced DNA damage blocks DNA synthesis during the polymerase chain reaction (PCR), thereby reducing the amount of amplified product of UV exposed DNA compared to control DNA. Thus, DNA lesions are indirectly quantified. This type of DNA dosimeter was first developed for monitoring the biologically effective DNA-damaging capacity of UVB doses integrated over time. The short or long-term effects of UVB doses can be obtained by varying the length of the DNA fragment to be analyzed by the PCR reaction [28,31].

Another type of DNA dosimeter that makes use of bacteriophage DNA is the phage T7 dosimeter [32]. For measuring DNA damage, a quantitative polymerase chain reaction (QPCR) methodology was developed using 555 and 3,826 bp fragments of phage T7 DNA. Basically, this assay is the same as that described above, where photoproducts block DNA replication by *Taq* DNA polymerase, thereby reducing the amplification of a damaged DNA segment. In addition, by using this system, it is possible to determine the inactivation (killing) of a phage particle as a consequence of DNA damage induction after UV exposure [36,37]. The calculation of the biologically effective dose (BED) is proportional to the inactivation rate [ln(n/n_0_)], where n_0_ and n are the number of active phages without irradiation and after UV exposure, respectively, thus corresponding to the average amount of UV damage in one phage particle. Consequently, the unit dose for phage T7 is defined by a survival rate of e^−1^ or, in other words, an average of one unit of lethal damage per phage particle. The number of active phages is determined by using *E. coli* B host cells through the plaque counting assay [36].

Although uracil is a component of ribonucleic acid (RNA), the uracil thin layer dosimeter is included within this category of biological dosimetry, for means of comparison of this methodology to the other DNA dosimeters described here. Both the structure and conformation of uracil bases in the polycrystalline form of uracil are suitable for forming cyclobutane type pyrimidine dimers through the photodimerization of uracil monomers [25]. Hence, uracil thin layers can be used as a nucleic acid model, when considering UV damage induction [27,38]. The UV radiation effect on these layers can be measured by the decrease in absorbance at the characteristic absorption band of uracil, whence the use of the OD (optical density) value at 288 nm for quantifying UV damage after biodosimeter exposure to various sources of UV radiation [38].

There are also DNA dosimeters based on the exposure of naked DNA solutions to sunlight. In one of the examples, a naked calf thymus DNA solution (10 mg L^−1^), stowed in cylindrical quartz tubes, was exposed to ambient solar radiation in Antarctica from October to December, 1998, for 3 h daily (12.00–15.00 h) during the UVB radiation-peak. The induction of CPDs was detected through the use of a specific antibody against this type of UV photoproduct. The results could be related to cloud-cover, ozone-column depth and spectrophotometric measurements of solar UV radiation. In short, subtle changes in solar spectral characteristics caused by ozone depletion could be detected with this biodosimeter. The highest CPD concentrations were observed when ozone-mediated shifts favored the shorter wavelengths of UVB radiation [30]. Actually, in the same year, another research work, also applying a naked calf-thymus DNA solution in quartz tubes, was published simultaneously and in the same volume of the journal. This DNA dosimeter was complemented with a phage dosimeter consisting of intact bacteriophage PWH3a-P1, which infects the heterotrophic bacterium *Vibrio natriegens*, thus facilitating the quantification of infectivity efficiency after exposure to sunlight. The viral and DNA dosimeters were applied together, whereupon a strong correlation was observed between dimer formation and the decay rates of viral infectivity, in accordance with increasing penetration of UVB radiation into the water column in the western Gulf of Mexico [39].

Our group also developed a suitable DNA-dosimeter system based on the exposure of a plasmid DNA solution (*pCMUT* vector) to artificial UV lamps and sunlight. In order to provide ample comprehension of the deleterious effects of solar UV radiation upon DNA molecules, different types of DNA damages (CPD, 6-4PP, and oxidized bases) were determined and quantified through the use of specific DNA repair enzymes and antibodies [6]. The biological effects of such lesions were also defined through the analysis of DNA inactivation rates and mutation frequencies, following replication of the damaged *pCMUT* vector in an *Escherichia coli MBL50* strain [15]. The most relevant results obtained with these very sensitive technologies, established the induction of CPD, as well as 6-4PP by UVA wavelengths. In order to demonstrate the biological effects of these DNA damages, mutagenesis and DNA inactivation were directly associated to the formation of large distorting DNA lesions, such as CPDs. These effects were not associated to the induction of oxidatively generated damage, independent of the UV wavelength applied (UVC, UVB, UVA, and sunlight) [6,15].

**Figure 5.**
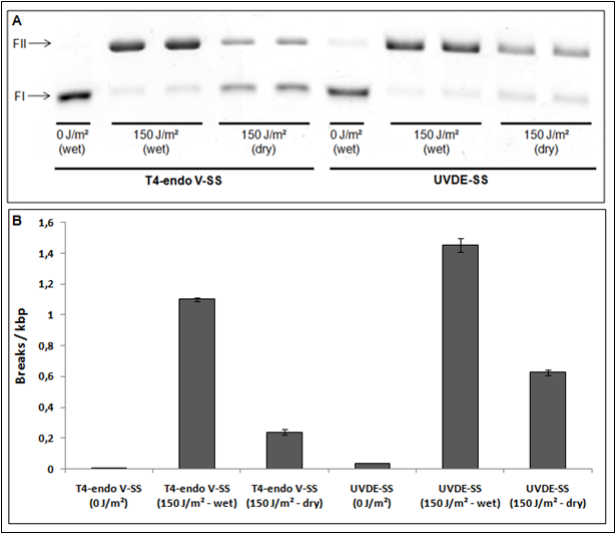
Analysis of UVC-induced DNA lesions induced in DNA in a buffer or under dry conditions. (**A**) Representative example of DNA photolesion induction after DNA exposure to UVC radiation. Plasmid DNA samples were UVC-exposed either diluted in a TE buffer (wet) or dried on a glass surface (dry) at room temperature and atmospheric pressure. 200 ng of both recovered DNA samples were treated with T4-endo V and UVDE enzymes. FI indicates the supercoiled DNA form and FII the relaxed DNA form resulting from enzymatic cleavage of DNA photoproducts. (**B**) Quantification of DNA photoproducts after UVC lamp exposure. T4-endo V-SS—T4-endonuclease V sensitive sites; UVDE-SS—Ultraviolet Damage Endonuclease sensitive sites (for details of the methodology employed the reader should refer to [6]).

Extremely important information regarding DNA dosimetry is the manner in which DNA samples are exposed to UV radiation. Experiments performed in our lab indicated that the efficiency of UV photoproduct induction, mainly CPD and 6-4PP, depends very much on the way irradiation is being carried out, in other words, either with the DNA sample diluted in a water/buffer solution or dehydrated thus forming thin layers on a surface. Furthermore, a previous work demonstrated that UVC irradiation carried out with a DNA sample in the dry state resulted in the formation of spore photoproducts, besides CPD and 6-4PP [13]. The induction of this specific UV photoproduct, noted in spores of certain bacteria, is not observed when DNA is irradiated in its physiological aqueous environment within all other types of cells. Therefore, a comparison was made of the induction of T4-endonuclease V-sensitive sites (T4-endo V-SS correspond to CPD) and Ultraviolet Damage Endonuclease-sensitive sites (UVDE-SS correspond to CPD, 6-4PP and other distorting DNA lesions), in DNA samples exposed to UVC light, in both the dry state and diluted in a buffer solution (TE buffer-10 mM Tris-HCl, 1 mM EDTA [pH 8.0]). The results are presented in Figure 5.

As shown, the induction of these lesions readily decreases when DNA samples are exposed to UVC light under dry conditions, when compared to samples maintained in a buffer during irradiation, thus indicating lower frequencies of photoproduct production under dry than wet conditions. Furthermore, the induction of T4-endo V-SS was 4.6-fold higher in the wet state than the dry, while the induction of UVDE-SS was only 2.3-fold higher, when so compared. Another important observation was that the ratios between the induction of putative CPDs and 6-4PPs (T4-endo V-SS/UVDE-SS-T4-endo V-SS) in the wet and dry states were 3.1 and 0.6, respectively. This implies the formation of photoproducts different from CPD or 6-4PP in DNA samples irradiated in the dry state, and which could be recognized by UVDE, thus decreasing the above ratio. Although the chemical analysis of this damage was not undertaken, it is presumed to be a spore photoproduct. The use of specific antibodies against CPD and 6-4PP would also help to better elucidate this question. Altogether, answers to these questions are important to understand mechanisms of UV-induced DNA damage, when this molecule is irradiated in dry conditions. Interestingly, a previous work had observed an important increase in the formation of inter-strand photoproducts when DNA is irradiated in the A-conformation, which would be predominant when this molecule is dried. In these conditions 6-4PPs were also detected in UVC irradiated DNA [40].

### 4.2. Spore Dosimetry

A spore dosimeter was developed, as a prototype biosensor for defining the DNA damaging capacity of UV irradiation. Biological measurements of solar UV irradiation using this biological system have been under way since 1999, at more than 20 sites in Asia, Europe and South America [41]. This biodosimeter reveals several features that make it suitable for worldwide comparison and long-term monitoring. It is based on the measurement of spore inactivation, when using highly UV-sensitive spores of a mutant strain of *Bacillus subtilis*, defective in both nucleotide excision repair and spore-photoproduct lyase [42,43]. The mutated spores are irradiated, spotted and dried on membrane filters. The greater part of inactivation is probably due to the formation of spore photoproducts (5-thyminyl-5,6-dihydrothymine). The spore inactivation dose can be calculated from the absolute value of the natural logarithm of the surviving fraction: SID = −ln(Ne/Nc), Ne and Nc being the average of colony-formers recovered from exposed and control spots [23]. Results reported in the literature demonstrate the usefulness of spore dosimetry in the continuous long-term measurement of biologically effective solar-UV irradiation at different latitudes [23,41,44]. Moreover, other additional works indicate this biological system to be one of the most versatile and convenient approaches to monitor human personal exposure to sunlight [33–45].

Similar to the spore dosimeter described above, another type of biological UV dosimeter, also employing *B. subtilis* spores, is the DLR-biofilm. In this case, an appropriate strain of *B. subtilis* needs to be chosen, depending on the dosimetric requirements of individual measurement. DNA-repair deficient strains can be used in DLR-biofilm dosimeters for short-term measurements (≥10 min), whereas DNA-repair wildtype strains are used for longer-term exposures (≤2 months). In general, DLR-biofilms are exposed in different types of exposure-boxes, with some areas remaining unexposed, to thus serve as dark controls during the period of exposure. The biofilm exposure housings are made of plastic and contain a spore biofilm in a water-tight biofilmstack. The DLR-biofilm method has also been adapted for application as a personal UV dosimeter [22].

### 4.3. Mammalian Cell Dosimetry

Compared to the other types of biological dosimeters, there is little information in the literature on systems that use mammalian cells as biodosimeters. On the other hand, there are several works reporting the use of efficient methodologies to quantify the hazardous effects of UV radiation upon these cells. An example worth mentioning is a rapid and convenient assay for the measurement of DNA damage and repair in specific genes using quantitative polymerase chain reaction (QPCR) of fragments from human genomic DNA after exposure to UVC light [46]. The same methodology, although in a different model, was applied to characterize the repair of DNA damage induced by UVC radiation in *C. elegans* [47]. Another highly accurate and quantitative assay that can be included here is based on HPLC coupled with tandem mass spectrometry. Through this approach, it was possible to assess the repair of the main photolesions in primary cultures of human keratinocytes [12] and to determine the type and the yield of formation of DNA damage in whole human skin exposed to UVB or UVA [10].

Nevertheless, in the higher organisms, complex responses, such as immunosuppression, tumor promotion, virus induction and photocarcinogenesis, require consideration after UV exposure [48–52]. Consequently, the use of laboratory animals, or at least human or animal cell-cultures, thereby replacing animal tests in biomedical research, is of great interest [53]. With this in mind, a biological UV dosimeter using rodent cells (RoDos) has been developed. The cells involved are Chinese hamster ovary cells AA8 (ATCC CRL-1859, repair proficient) and UV5 (ATCC CRL-1865, defective in nucleotide excision repair gene ERCC2). The RoDos dosimeter comprises two parts: (i) Rodent cells growing on a UV-transparent petriPERM^®^ foil. (ii) A special device, which facilitates exposure of growing cells to different doses of UV light, and the cultivation of cells in exposed and unexposed areas of the petriPERM^®^ dish under identical conditions. The influence of UV exposure on cell growth is determined by image analysis, involving the correlations of optical densities (OD) of irradiated to unirradiated areas. It is suitable for evaluating the cytotoxic effects of simulated sunlight, as well as characterizing UV sources and the protective capacity of sunscreens [35].

Another different *in vitro* system is used as a photobiological tool for evaluating the molecular response of simulated sunlight upon human keratinocytes growing as monolayers or as part of reconstructed skin. In fact, although not strictly a biological dosimeter, it can also be applied when assessing the photoprotection of sunscreens associated with DNA photodamage, whereat the formation of single strand breaks (SSB) and CPDs by comet assaying, and the induction of transcription factor p53, are the main parameters considered. These models are suitable in the development of quantitative methodologies for use as alternative *in vivo* tests, when assessing the photoprotective efficacy of sunscreens [54].

Furthermore, and also for evaluation of sunscreens, another *in vitro* model of reconstructed skin has been developed for exposures to simulated daily irradiation with or without photoprotection. The biological effects induced after irradiation in a solar simulator are assessed by the histology of artificial skin, vimentin immunostaining for dermal fibroblasts, and the analysis of matrix metalloproteinase (MMP)-1 secretion. On considering these evaluated endpoints, it has been suggested that sunscreen ingredients should be better balanced with an adequate level of UVA absorption, to thus ensure efficient daily photoprotection. There are also indications that higher SPF (Sun Protection Factor) values were not proportionally paralleled by the UVA screening capacity of the formulation [55].

### 4.4. Vitamin D Dosimetry

Contrary to most biological dosimeters that incorporate DNA sensitivity to the damaging effects from UV radiation, the process of vitamin D synthesis is beneficial by nature. In fact, vitamin D synthesis, the most well-known and well-documented beneficial effect of solar UVB irradiation, consists of two basic stages, *viz.*, the photosynthesis of previtamin D (provitamin D photoisomerization), and its thermoconversion into vitamin D. The former constitutes the base of Vitamin D dosimetry. The kinetics of previtamin D accumulation is intimately related to the endpoint of the reaction in vitamin D synthesis, and represents the biological effect under study in this case [26].

The vitamin D dosimeter uses a low-concentrated solution of provitamin D (ergosterol or 7-dehydrocholesterol), diluted in ethanol (C = 0.002%) and stored in quartz cuvettes for UV irradiation. Before exposure to sunlight the absorption spectrum is recorded by a spectrophotometer within the 230–330 nm range. The daily accumulated dose is measured through daytime exposure of the solution, with hourly absorption-spectra monitoring. In fact, the values of provitamin D-previtamin D photoconversion are accompanied by absorption spectrum changes, with the OD decreasing at 282 nm. Thus, through optical analysis, a function of the exposure time can be evaluated in order to determine the biologically effective dose of sunlight [26].

## 5. Conclusions and Perspectives

In a scenario of uncertainty as to the effects of climate change upon the incidence of solar UV irradiation, the application of biosensors parallel to physical photometry will be of aid in obtaining important information for the future protection of ecosystems and human health. The biosensors described in this work are compared in Table 1.

Many are portable and easy to use, but yield information limited to the effects on the molecules themselves, while others allow indication of biological activities. The use of mammalian cells consists, obviously, on measurements closer to the effects in human, but these cells are more difficult to handle. Notwithstanding, further development in mammalian-cell biodosimeters is still a basic requirement for biomedical studies. In this sense, it is interesting to mention human patients bearing the xeroderma pigmentosum (XP) syndrome, an autosomal recessive genetic disorder that is clinically characterized by the increased frequency of skin cancer in those regions of the body that are normally exposed to sunlight. Fibroblasts from these patients, besides being very sensitive to UV, are defective in their capacity to remove (nucleotide excision repair) or to replicate (translesion synthesis) UV-induced DNA photoproducts [56]. An interesting approach to obtain sensitive and biologically relevant information on sunlight deleterious action would be by using of XP derived DNA repair-deficient human-skin cells. These cells could constitute a powerful tool for assessing the harmful effects of sunlight on human genomic DNA, and the consequences arising from the induction of different processes of cell-death and mutagenesis. Moreover, the development of biological dosimeters based on the cells from these patients could complement the evaluation of the photoprotection potential of sunscreens, and be of aid in the development of new and more efficient UV-protecting products focused on the specific needs of this group of people.

In another approach, one of the most exciting promises in this field is the application of biological dosimetry to astrobiological exploration programs. The proposed experiments would provide tools for the scientific investigation of those processes involved in the birth and evolution of life on Earth, besides possibly demonstrating the importance of protecting the Earth’s future environment from anthropogenic emission of destructive gases that could compromise the ozone layer. Most of the biodosimeters described here, *viz.*, DNA-dosimeters [57,58], uracil thin layers [38], and spore dosimeters [59–61], are potentially applicable in astrobiological studies.

Thus, novel UV-sensors based on biological models are an increasing requirement for an enormous range of applications, and continuous efforts in scientific and technological research are essential for a better comprehension of the expected environmental changes.

## Figures and Tables

**Table 1.** Comparison of different biosensors.

**Method [reference]**	**Sensitivity (minimal UV dose)**	**Endpoint analysed**	**Approach**	**Positive Features**

Vitamin D [26]	40.0 J/m^2^ (282 nm)	Photoisomer concentration	Spectrophotometric	Chemical measurements, analysis a beneficial effect, easy to perform, high spectral selectivity.
Uracil thin layer-OD [24]	10.0 J/m^2^ (254 nm)	Polycrystalline uracil thin-layer	Spectrophotometric	Chemical measurements, easy to perform.
Uracil thin layer-OWLS [27]	0.8 J/m^2^ (254 nm)	Optogeometrical parameters of a thin layer	Refractive index changes	Chemical measurements, easy to perform.
Bacteriophage λ DNA [31]	6.1 kJ/m^2^ (sunlight)	DNA polymerase blockage	PCR	Portable, robust, stability.
Phage T7 DNA [32]	10.0 J/m^2^ (254 nm)	DNA polymerase blockage	QPCR	Portable, robust, stability.
Naked calf thymus DNA solution [62]	∼10.0 J/m^2^ (equivalent to UVB)	CPDs	Antibodies	Portable, direct lesion measurements.
Naked calf-thymus DNA + bacteriophage PWH3a-P1 [39]	1.9 kJ/m^2^ (305 nm) 41.0 kJ/m^2^ (320 nm)	CPDs, plaque forming units	Radioimmunoassay, viral infectivity	Portable, direct lesion measurements, determines biological activity.
Plasmid DNA [6]	50.0 J/m^2^ (UVC) 2.0 kJ/m^2^ (UVB) 50.0 kJ/m^2^ (UVA)	CPDs, (6-4)PPs, oxidized bases, SSBs, plasmid viability	DNA repair enzymes, antibodies, alkali treatment, genotoxic effects	Portable, robust, direct and specific lesions measurements, determines biological activity.
*B. subtilis* spore [41]	650.0 J/m^2^ (sunlight)	Colony forming units	Spore inactivation	Portable, robust, easy to perform, determines biological activity.
DLR-biofilm, *B.subtilis* spore [22]	10.0 J/m^2^ (254 nm)	Optical density	Image analysis	Portable, robust, easy to perform.
RoDos [63]	1.0 J/m^2^ (UVC)	Colonies, optical density	Cellular survival, image analysis	Direct evaluation of biological effects on mammalian cells
Human keratinocytes [54]	3.5 kJ/m^2^ (UVB) 42.7 kJ/m^2^ (UVA)	CPDs, SSBs	Comet assay, antibodies	Direct evaluation of biological effects on human cells
Reconstructed skin [54]	3.5 kJ/m^2^ (UVB) 42.7 kJ/m^2^ (UVA)	CPDs, SSBs	Comet assay, antibodies	Direct evaluation of biological effects on human cells
